# Adaptation and uptake evaluation of an SMS text message smoking cessation programme (MiQuit) for use in antenatal care

**DOI:** 10.1136/bmjopen-2015-008871

**Published:** 2015-10-22

**Authors:** Felix Naughton, Sue Cooper, Katharine Bowker, Katarzyna Campbell, Stephen Sutton, Jo Leonardi-Bee, Melanie Sloan, Tim Coleman

**Affiliations:** 1 Behavioural Science Group, Institute of Public Health, University of Cambridge, Cambridge, UK; 2 Division of Primary Care, U.K. Centre for Tobacco and Alcohol Studies and National Institute for Health Research School for Primary Care Research, University of Nottingham, Nottingham, UK

**Keywords:** smoking cessation, pregnancy, text message, service evaluation

## Abstract

**Objectives:**

To adapt a tailored short message service (SMS) text message smoking cessation intervention (MiQuit) for use without active health professional endorsement in routine antenatal care settings, to estimate ‘real-world’ uptake and test the feasibility of its use.

**Design:**

Single-site service evaluation.

**Setting:**

A Nottinghamshire (UK) antenatal clinic.

**Participants:**

Pregnant women accessing the antenatal clinic (N=1750) over 6 months.

**Intervention:**

A single-sheet A5 leaflet provided in the women's maternity notes folder describing the MiQuit text service. Similar materials were left on clinic desks and noticeboards.

**Outcome measures:**

MiQuit activation requests and system interactions were logged for two time frames: 6 months (strict) and 8 months (extended). Local hospital data were used to estimate the denominator of pregnant smokers exposed to the materials.

**Results:**

During the strict and extended time frames, 13 and 25 activation requests were received, representing 3% (95% CI 2% to 5%) and 4% (95% CI 3% to 6%) of estimated smokers, respectively. Only 11 (44%) of the 25 requesting activation sent a correctly formatted initiation text. Of those activating MiQuit, and invited to complete tailoring questions (used to tailor support), 6 (67%) completed all 12 questions by text or website and 5 (56%) texted a quit date to the system. Of the 11 activating MiQuit, 5 (45%, 95% CI 21% to 72%) stopped the programme prematurely.

**Conclusions:**

A low-intensity, cheap cessation intervention promoted at very low cost, resulted in a small but potentially impactful uptake rate by pregnant smokers.

**Strengths and limitations of this study**

- This is the first ‘real-world’ uptake evaluation of a text message smoking cessation intervention for pregnant smokers.
- The low cost of the promotional materials and the text message smoking cessation intervention they promoted mean that even a very small uptake would likely confer significant public health impact if intervention effectiveness were demonstrated.
- Estimating the denominator of individuals exposed to promotional materials in a real-world context to calculate uptake is challenging and reliance on routinely collected National Health Service data may have generated bias.

## Introduction

Prenatal smoking increases the risk of miscarriage, stillbirth, preterm birth, low birth weight, neonatal mortality,^1^ ^2^ asthma^3^ and childhood obesity^4^ and is estimated to cost the UK National Health Service (NHS) between £20 and £87.5 million annually.^5^ Supporting pregnant women to quit smoking remains a public health priority.

In England, routine care for pregnant women identified as smokers is a referral for support to Stop Smoking Services for Pregnant women (SSSP). The SSSP typically provide face-to-face behavioural support in combination with nicotine-replacement therapy with follow-up visits or phone calls.^6^ However, only around 15% of pregnant smokers access these.^7^ Given that the majority of pregnant smokers are interested in receiving help to quit smoking^8^ but only a minority engage with the SSSP, there is an urgent need to develop new, effective cessation support options that are sufficiently used by pregnant smokers to confer a public health benefit.

One potential form of additional cessation support that could be routinely offered in antenatal care is self-help. A meta-analysis of self-help intervention trials found that self-help almost doubles the chances of abstinence among pregnant smokers compared with usual care.^9^ Furthermore, self-help is of high interest to pregnant smokers.^8^ A delivery medium showing promise for delivering self-help to pregnant smokers is text messaging.^10^ Text messaging is cheap and could easily reach many pregnant smokers as most have mobile phones.^11^ Also, among non-pregnant smokers, text message-based cessation support is effective.^12^

We have developed an individually tailored smoking cessation text messaging intervention for use in pregnancy, called MiQuit; in a small randomised control trial (RCT) this was found feasible to deliver, acceptable to pregnant smokers and also, potentially effective.^10^

While clinical trials can establish the effectiveness of public health interventions, the impact of an intervention is a function of its effectiveness multiplied by ‘real-world’ uptake. Therefore, for an intervention such as MiQuit, which is very unlikely to harm patients, establishing its likely use should be part of the evaluative process that determines whether or not a large scale efficacy RCT should be undertaken. For example, if in ‘real-world’ settings almost no pregnant smokers could be persuaded to use MiQuit, then an expensive, large RCT would be unnecessary. However, as MiQuit is so inexpensive to deliver, if even a small proportion of pregnant smokers used it, then a large, definitive RCT would be much more justifiable as demonstrating even modest efficacy would likely ensure that MiQuit is judged cost-effective.

Since the feasibility trial,^10^ we have modified MiQuit so that it now should be possible for smokers to initiate this themselves, without assistance. This modified MiQuit intervention was made available, without introduction from health professionals, to all pregnant women attending one antenatal clinic for antenatal care. In this paper we describe MiQuit modification and distribution processes and assess the feasibility of women's ‘self-initiation’ of the programme which involves their completion of ‘tailoring’ questions via text message or a website. We also estimate pregnant smokers’ rates of initiating and subsequently cancelling MiQuit, and evaluate these in the context of pregnant smokers’ engagement with local NHS SSSP.

## Methods

### Design

This was a single-site service evaluation (King's Mill Hospital (KMH), Sherwood Forest Hospitals NHS Foundation Trust) where initiation and subsequent discontinuation rates for a smoking cessation text message support system (MiQuit) were recorded among a cohort of pregnant women. The National Research Ethics Central Queries Service confirmed that no ethical review was required as this was a service evaluation. The investigation was conducted in accordance with the ethical principles that have their origin in the Declaration of Helsinki, 1996.

### Adaptations to MiQuit for use in routine care

The MiQuit text message cessation support system was initially developed for use within evaluation research and its original development has been described in detail elsewhere.^10^ MiQuit delivers a 12-week advice and support programme on quitting smoking in pregnancy, using participant characteristics to tailor the support to each individual. Most of the support delivered is part of a structured delivery schedule of either 0, 1 or 2 texts per day. The frequency of scheduled messages is highest in the first 4 weeks and then reduces for the remainder of the programme. During the programme, users are sent two texts enquiring as to whether or not they had smoked in the previous 3 days (at 3 and 7 weeks). Responses to these assessment messages are primarily used to tailor the support subsequently delivered but they also aim to increase engagement. Additionally, system users can request support on demand by texting the system one of two keywords: HELP if they are experiencing a craving or temptation to smoke and SLIP if they have smoked during a quit attempt.

The original system required tailoring characteristics to be collected by paper questionnaire or telephone after which researchers initiated the support system manually. For this study, to ensure MiQuit could be used in routine care without the need for interpersonal communication, we needed to make two main adaptations to the initiation procedure (table 1): (1) setting up an automatic support initiation mechanism that allows individuals to text a key word (QUIT) plus their first name and current number of weeks of gestation (eg, ‘quit Katie 13’) to a six-digit shortcode number to initiate MiQuit support and (2) setting up an automated procedure for collecting information about each individual to enable the support to be tailored. To achieve this, we reduced the number of tailoring characteristics from 26 to 12 to minimise response burden. These 12 characteristics were those considered of greatest clinical importance that demonstrated sufficient variability in response to enable support to be meaningfully tailored. These characteristics included motivation to quit smoking, the hardest situation to avoid smoking, cessation self-efficacy, the number of cigarettes smoked per day, time to first cigarette after waking, partner's smoking status and reasons for quitting. We set up 12 pairs of automated ‘question-and-response’ text messages that invited users to text answers to tailoring questions. In case of non-response, we set up 24 h reminders for each question with the option to skip a question according to user preference. In addition, we set up a website (http://www.miquit.co.uk) to enable all users to complete questions online if desired and in case they incurred a charge for sending text messages. We also enabled the system to ignore any tailoring variable if that variable was missing and instead use the median response for that question from the previous feasibility trial.^10^ As a consequence, if no tailoring questions are answered the system delivers entirely generic support, apart from the use of the individual’s name and weeks of gestation.

**Table 1:** Summary of adaptations made to MiQuit for use in ‘real-world’ settings

MiQuit—used in feasibility trial	MiQuit—adapted for use in routine care
*Initiation procedure*	
Initiation manually activated by researchers	Users text QUIT plus their first name and weeks of gestation (‘quit Katie 12’) to a six-digit shortcode
Tailoring data (26 characteristics) collected by a paper questionnaire or completed over the phone	Tailoring data (12 characteristics) collected via automated multiple choice text message questions or website
*Increasing support coverage and quality*	
N/A	Messages mapped onto BCTs appropriate to intervention objectives. New messages created where coverage was low
N/A	Messages were reviewed by two smoking cessation experts to ensure accuracy and quality of content and refinements made in response to feedback
*Interactivity*	
On-demand support if users are experiencing a craving to smoke (text HELP) or if they have smoked during a quit attempt (text SLIP)	Addition of the keyword QUIZ to play a multiple choice text message trivia quiz for distraction
Smoking status assessment text messages sent at 3 and 7 weeks into programme to increase engagement and further tailor the support delivered	Addition of an end of programme (12 weeks) smoking status assessment text message
N/A	Facility created where users can increase or decrease frequency of support by texting MORE or LESS
N/A	Facility created to provide an additional schedule of support messages if user texts in a quit date
N/A	Series of gestation-tailored baby development information texts
N/A	Additional smoking in pregnancy risk information messages only delivered if user responds to an invitation prompt text

BCTs, behaviour change techniques.

Further adaptations were made to increase cessation support coverage, quality and interactivity (table 1). These adaptations were informed by qualitative studies,^13^ ^14^ a feasibility trial,^10^ a smoking cessation-specific taxonomy of behaviour change techniques (BCTs),^15^ expert consultation and further piloting: (1) existing messages were mapped onto smoking cessation BCTs and new messages created where there was low coverage of BCTs that aligned with MiQuit intervention objectives; (2) refinements were made to the content of messages relating to accuracy and quality in response to feedback from experts in smoking cessation; (3) we added the keyword QUIZ for on-demand support, enabling users to play a multiple choice text message trivia quiz game designed to support distraction from smoking; (4) at the end of the programme (12 weeks), an additional smoking status assessment message was added; (5) a facility for enabling users to increase or decrease the frequency of support texts at any time was developed by texting MORE or LESS; (6) we incorporated the facility for users to text in a quit date to receive an additional 10-day schedule of text support orientated around this date, which could be reset at any time; (7) we added a series of gestation-tailored baby development information texts sent every 2 weeks during the programme to promote a mother-orientated identity and (8) we also added a set of smoking in pregnancy risk information messages that were only delivered if the user replied to an invitation prompt text.

### Participants

Pregnant women attending a booking visit with their community midwife from KMH between January and September 2013.

### Intervention and procedure

All pregnant women in England receive a maternity notes folder at their first ‘booking’ appointment with their midwife at approximately 8–12 weeks gestation. These notes are held by the mother throughout her pregnancy and include her personal maternity records from all antenatal appointments. A single-sheet A5 leaflet describing MiQuit was inserted into 1750 of these maternity notes folders within the antenatal clinic in mid-December 2012. Midwifery management indicated that it would take several weeks before these maternity notes folders reached circulation. Based on hospital historical data it was estimated that this number of leaflets would cover approximately 6 months of booking appointments by community midwives. The promotional leaflet was inserted alongside two other health advice leaflets not related to smoking. In addition to providing information about the support provided and potential costs associated with activating MiQuit, the leaflet explained how to activate MiQuit support (see online supplementary material) and how to discontinue text support. Similar MiQuit information was included on A3 posters placed in the ultrasound clinic area and the antenatal ward and promotional ‘banner’ pens and credit card-sized information cards which were left in the ultrasound clinic area within the antenatal clinic. As our intention was to estimate the rate of initiation of text message support in the absence of health professionals’ recommendations to do so, maternity staff were not directly informed about the promotional materials. Checks were made with maternity managers in March 2013 to ensure promotional materials were made available as expected.

### Initiating MiQuit

After reading the advertising leaflet, a woman could initiate MiQuit by sending a text message to the shortcode number listed; this text either cost the user their standard rate for sending text messages (usually between 8 and 12 pence) or was free, depending on their mobile provider/tariff. All subsequent text messages sent to MiQuit were sent to a ‘virtual reply number’ (VRN), the equivalent of sending a text message to a UK mobile phone. Completion of tailoring questions using the website was free, unless users were charged by their provider for internet usage. To discontinue the support at any stage, users were advised to text STOP to the VRN.

### Measures

The outcome measures were estimates of the proportions requesting MiQuit support activation among (1) pregnant women and (2) pregnant smokers. The proportion of those who activated the programme who subsequently discontinued it prematurely by texting STOP was also collected, by the MiQuit system. Feasibility measures included correct use of the incoming activation request text messages and the number of tailoring questions completed. General system usage data was also collected. Nicotine dependence was measured using an adapted version of the Heaviness of Smoking Index,^16^ collected as part of the tailoring questions.

Rates of access to the local Stop Smoking Service for Pregnant women (SSSP), Nottinghamshire New Leaf, were calculated using routinely collected data from the Hospital Trust and the SSSP. Access was defined as those pregnant smokers attending at least one smoking cessation appointment. We collected these data to help assess the rate of MiQuit activation in the context of rates of other use of cessation support, without any prior hypothesis that one should affect the other.

### Sample size

Using historical data from the KMH, during the 12-month period April 2011 to March 2012, 894 babies were born to women who had been recorded as smoking at booking. Therefore, during the 6 months when MiQuit would be made available, we estimated that approximately 450 women who smoke would attend a booking appointment for their pregnancy and receive a MiQuit promotional leaflet. With this sample, an uptake rate of 5% would be estimated with 95% CIs of ±2% (3% to 7%) and of 10% with 95% CIs of ±3% (7% to 13%).

A priori, it was decided that MiQuit activation requests would be monitored only for the 6-month period when promotional materials were intended to be made available (‘strict period’). However, a significant number of activation requests occurred during the subsequent 3 months, due most likely to a lag in the booking packs that contained the materials being handed out by midwives, supplementary materials still in circulation or to women retaining the materials and taking a short while to decide to activate MiQuit support. Site visits confirmed that the booking notes folders containing the MiQuit leaflets had all been taken by midwives by mid-June 2013 and the information cards and pens had been removed from the antenatal clinic by the beginning of August, but may have been available until then. Therefore, *post hoc,* an ‘extended period’ for activations, potentially reflecting a more realistic scenario, was used for comparison. This used all activation requests recorded for the period when activations were logged (approximately 9 months), using an 8-month period (January–August 2013) as the period of availability of materials that is, when pregnant women had direct access to the materials. MiQuit was not promoted outside of this evaluation and individuals could only sign up using the shortcode and keyword combination unique to this study.

### Statistical analysis

To estimate the proportion of pregnant women and pregnant smokers who requested MiQuit support out of those exposed to promotional materials, we used the number of activation requests during the two time periods with equivalent estimated denominators of all pregnant women and pregnant smokers using KMH data. Smokers were defined as those reporting smoking at booking. The proportion of individuals sending an activation request and discontinuing MiQuit are presented with 95% CIs using the Wilson score method without continuity correction due to the small expected event rate.

To estimate the equivalent SSSP access rate of women referred from KMH, the number of pregnant smokers identified at booking for the whole Trust was divided by the number of pregnant women who attended an appointment with the local SSSP who were referred from the Trust. The proportion of women referred from KMH out of those from the Trust was then calculated and applied to calculate the access rate.

## Results

For the 6-month (January–June 2013) and 8-month (January–August 2013) periods when promotional materials were available, 1775 and 2356 women, respectively, were booked for antenatal care with the Kings Mill Trust. Among the women attending these appointments, there were 449 smokers recorded (25.3%) in the 6-month and 585 smokers recorded (24.8%) in the 8-month periods. During the strict 6-month period, 13 activation requests for MiQuit were received representing 0.7% (95% CI 0.4% to 1.3%) of all pregnant women and 3% (95% CI 2% to 5%) of those estimated to be smokers. For the extended 9-month period for activations, 25 requests were received representing 1.1% (95% CI 0.7% to 1.6%) of all pregnant women and 4% (95% CI 3% to 6%) of estimated smokers (table 2).

**Table 2:** Estimated uptake rates of MiQuit among pregnant women and pregnant smokers using two time period scenarios at the King's Mill Hospital (2013)

Scenario	Material availability period	Activation period	Maternity bookings	Smokers at booking (%)	Activation requests	Uptake estimate for pregnant women (95% CI)	Uptake estimate for pregnant smokers (95% CI)
Strict	6 months (January–June)	6 months (January–June)	1775	449 (25.3)	13	0.7% (0.4% to 1.3%)	2.9% (1.7% to 4.9%)
Extended	8 months (January–August)	9 months (January–September)	2356	585 (24.8)	25	1.1% (0.7% to 1.6%)	4.3% (2.9% to 6.2%)

Of the 25 activation requests received for MiQuit within the study duration, only 11 (44%) successfully sent an activation text message which included the requested information and consequently activated MiQuit support. Of those who did not send the requested information, 11 (44%) sent just the keyword QUIT and 3 (12%) sent QUIT and their name and weeks of gestation, but in a format that was not readable by the system.

Of the 11 who activated MiQuit support, 5 (45%, 95% CI 21% to 72%) discontinued the programme prematurely by texting STOP. Two of these individuals texted STOP shortly after activating the support and the remaining three, who all indicated that they were currently smoking in response to the 3-week smoking status assessment message, stopped the support shortly afterwards. The timing of the STOP requests from these three individuals corresponded closely with a scheduled text message reminding users how to stop the support.

The mean gestation of the 11 individuals who activated MiQuit support was 15 (SD=9.2) weeks. In terms of trimester, five (46%) were in the first, four (36%) in the second and two (18%) in the third trimester. Nine individuals remained in the programme long enough to be invited to complete tailoring questions. Six (67%) completed all tailoring questions, three via text messages and three via the website. The remaining three individuals responded to 1, 2 and 8 tailoring questions, respectively. Five (56%) texted a quit date to MiQuit and two (22%) increased the intensity of the text message support with no users decreasing the intensity. Of those providing sufficient information for calculating nicotine dependence (n=7), two users (29%) had high dependence, three (43%) had medium and two (29%) users had low dependence.

Equivalent access rates for the local Stop Smoking Service for the extended period were 5% (95% CI 4% to 6%) of all pregnant women and 17% (95% CI 14% to 19%) of estimated pregnant smokers.

## Discussion

When an short message service (SMS) text message smoking cessation support programme (MiQuit) was adapted for use in routine care and made available to all pregnant women in one NHS Trust using a basic method of promotion without active recommendation from health professionals, an estimated 3–4% of pregnant smokers requested to initiate use. While difficulties were experienced in sending the initiation text message in the correct format, the majority of those who successfully initiated MiQuit completed all 12 tailoring questions and almost half texted in a quit date to the system.

As far as the authors are aware, this is the first ‘real-world’ uptake evaluation of a text message smoking cessation service among smokers and the first investigation of non-routine cessation support uptake among pregnant smokers. The investigation provides an estimate of uptake for a very low-cost promotion of a cessation intervention that can be used without health professional instruction. We have demonstrated that a significant, though small, minority of pregnant smokers in one site were sufficiently interested in cessation support that they would activate self-help as a result of unsolicited promotional materials. Requests for MiQuit activation represent approximately 25% of those attending at least one face-to-face support consultation with the Stop Smoking Services. Real-world uptake estimates are valuable as they can be combined with an effectiveness estimate to allow a precise calculation of cost-effectiveness, which can inform commissioning decisions better than efficacy data alone.^17^

However, there are challenges with estimating real-world uptake, including limitations specific to this evaluation. A particular challenge was estimating the denominator of pregnant smokers. Our approach is open to error as (1) the time periods of the study and the collection of the smoking data do not correspond exactly and (2) the hospital data may underestimate the number of pregnant smokers who had engaged with maternity care due to misreporting.^18^ The latter bias, if present, could have resulted in an overestimation of the proportion of pregnant smokers who requested to initiate MiQuit. Conversely, both time frame scenarios used may have underestimated uptake. The first scenario did not allow for all women to delay uptake and the second included 25% of women in the denominator used to calculate uptake who may not have received the MiQuit leaflet, as their booking appointment was subsequent to all leaflets having been given out in the maternity notes folders. In rare cases women can be booked within an antenatal clinic with a booking notes pack from an outside clinic, which would underestimate uptake further. Another limitation is the difficulty in identifying which promotional materials motivated women to initiate support. While we consider the leaflet to have been the main source of information, supported by the findings from a small number of qualitative interviews undertaken among those who initiated support, the information cards, promotional pens and posters may have accounted for a significant proportion of the initiation requests. Furthermore, midwives may have become aware of the materials and promoted them directly, as found in one of the interviews. It is possible that materials placed within the antenatal clinic could have been seen by women in later pregnancy who might not have been included in the denominator of pregnant women because they entered antenatal care prior to January 2013.

We found an uptake rate for MiQuit which is very similar to that reported for telephone quitlines, viewed as central to tobacco control policy in countries such as Australia and the UK. For example, during promotional national mass media campaigns in Australia and England, access rates among general smokers for telephone quitlines were 3.6% and 4.2%, respectively.^19^ ^20^ Unlike the MiQuit uptake evaluation, not all access to the quitlines would have been attributable to the mass media campaigns and some calls would not have resulted in the receipt of formal smoking cessation support. Nevertheless, this level of engagement with quitlines is considered an important public health achievement.^19^ Recently, a national campaign to promote a self-help ‘Quit Kit’ in England including mass media, supermarket and Stop Smoking Services promotions resulted in an uptake of approximately 5% when applying a model of smoking behaviour in England.^21^ ^22^ The MiQuit uptake rate observed using a low-intensity approach could be considered to compare favourably to other more intensive and costly efforts to promote uptake of cessation support.

If demonstrated to be effective, in line with the effect estimates from a feasibility trial^10^ and similar text message interventions for non-pregnant smokers,^12^ the low cost of delivering MiQuit (approximately £3.20 per user based on trial data plus maintenance costs) and low dissemination costs to promote it would likely make it highly cost-effective. Delivering self-help support by text message could therefore make an important contribution to the reduction of maternal and infant complications and disease due to maternal smoking. Lessons learned from the current investigation include the importance of using a simple activation procedure given the high rate of activation failures and the consideration of using fewer than 12 tailoring questions when pregnant smokers initiate completion of these by text message rather than website.

Compared with a feasibility trial,^10^ we observed a markedly higher discontinuation rate (46% vs 9%). While this is similar to the 44% discontinuation rate of routine SSSP support, using a definition of those not attending a 4-week follow-up appointment,^7^ these rates may not be directly comparable given the difference in the type of support provided, effort required to discontinue them and reasons for doing so. Interestingly the MiQuit feasibility trial found that only 25% of participants discontinuing MiQuit support prematurely found the programme annoying.^10^ Further insight into rates of premature discontinuation of text message support in real-world contexts is therefore required. Of note is that non-pregnant smokers who discontinue cessation text message support are found to be more likely to have engaged in quitting smoking and subsequently relapsed than not have attempted to quit at all.^23^ Therefore, it cannot be assumed that discontinuation reflects lack of engagement with attempting to quit smoking. When using SSSP appointment rates to contextualise MiQuit uptake rates, our estimate of the proportion engaging in cessation support, 17%, is likely to have been an overestimate. A UK evaluation study of an ‘opt-out’ referral pathway, a procedure implemented at the KMH during the evaluation period, found that only 60% of women attending a smoking cessation appointment set a quit date; a prerequisite for receiving cessation support.^24^ Therefore, MiQuit uptake rates may be closer to the uptake of SSSP support than initially estimated.

Identifying how to optimise promotion and uptake of self-help interventions like MiQuit are important areas for future investigation. One important format to compare to the promotion approach used in this investigation is staff or health professional endorsement alongside the provision of promotional materials. Given the demonstration that unsolicited promotional materials appear sufficient on their own to generate uptake, the time cost of a health professional providing a leaflet to pregnant smokers would be low and could potentially increase cost-effectiveness if uptake was increased. Leaflet design is another aspect which could affect uptake including the use of NHS branding, which was not used in this investigation. Finally, it is important to establish whether uptake of text messaging support might decrease use of other cessation support. These remain areas for future research.

### Conclusion

Adapting a text message cessation service, originally designed for use in research, for use in routine care was feasible, and promoting its use without active recommendations from health professionals resulted in 3–4% of estimated pregnant smokers requesting to receive text message cessation support. Most individuals who initiated the text message support engaged with the request for smoking-related information in order to tailor the support, via text message or website. Almost half chose to discontinue the text message support before programme completion. However, further research is required to better understand the reasons why some recipients of text support discontinue it prematurely. These findings suggest that a large, definitive trial testing MiQuit efficacy would be a rational use of resource funds.
